# Inside out: a rare case of nonpuerperal uterine inversion caused by submucosal leiomyoma

**DOI:** 10.1093/jscr/rjaf803

**Published:** 2025-10-16

**Authors:** Andrew Pratama Kurniawan, David M Allorante

**Affiliations:** Department of Obstetrics and Gynecology, Cipto Mangunkusumo Hospital, Faculty of Medicine Universitas Indonesia, Jl. Salemba Raya No. 6, Kenari, Kec. Senen, Kota Jakarta Pusat, Daerah Khusus Ibukota, Jakarta 10430, Indonesia; Department of Obstetrics and Gynecology, Karawang General Hospital, Jl. Galuh Mas Raya No. 1, Sukaharja, Telukjambe Timur, Karawang, Jawa Barat 41361, Indonesia

**Keywords:** uterine inversion, nonpuerperal uterine inversion, uterine leiomyoma, vaginal mass, hysterectomy

## Abstract

Nonpuerperal uterine inversion (NPUI) is an extremely rare condition characterized by the uterus turning inside out through the endometrial cavity or cervix. Due to its rarity, NPUI presents diagnostic and therapeutic challenges, especially in atypical cases. This report discusses the management of such a case. A 50-year-old woman, P1A0, was referred with a protruding mass from her vagina. With no cervix part identified and no uterus found on abdominal ultrasound, uterine inversion with submucosal leiomyoma was diagnosed. Repositioning was done from the vagina and abdomen using a sondage. Total abdominal hysterectomy was performed. NPUI is a rare condition often caused by uterine fibroids, presenting with vaginal bleeding, pain, and a protruding mass. Diagnosis of uterine inversion relies on clinical signs, ultrasound, or magnetic resonance imaging could help but is not essential. Treatment depends on reproductive goals, but often requires hysterectomy following uterus repositioning. Conservative approaches are rare, and their impact on fertility remains uncertain.

## Introduction

Uterine inversion is a very rare case characterized by the inversion of the uterus inside out through the endometrial cavity or up to the cervix [1]. It happens most often after labor, or during the puerperial period. However, some cases are nonpuerperal/gynecological and unrelated to pregnancy, called “nonpuerperal uterine inversion” (NPUI) [2]. Although the mechanism is not very clear, the major risk factors involve mass in the uterine cavity, such as submucosal leiomyoma and sarcoma, fundal location of the tumor, a thin uterine wall, and cervical dilation [3].

The rare entity of NPUI poses a diagnostic and therapeutic challenge, especially in atypical cases. The presence of a palpable mass in the vaginal canal could mimic other benign gynecological conditions and could result in inadequate treatment decisions [4]. Prompt treatment and surgical intervention are essential to treat this problem. This case report would like to report the management of uterine inversion.

## Case presentation

A 50-year-old woman, P1A0, was referred with a protruding mass from her vagina since 2 days before admission. She was constipated before, and suddenly a mass was protruding from her vagina. The mass could not be reduced and was associated with difficulty emptying the bladder without any heavy bleeding. She denied prior uterine prolapse or urinary tract symptoms. Patient had uncontrolled diabetes as her past medical history.

**Figure 1:**
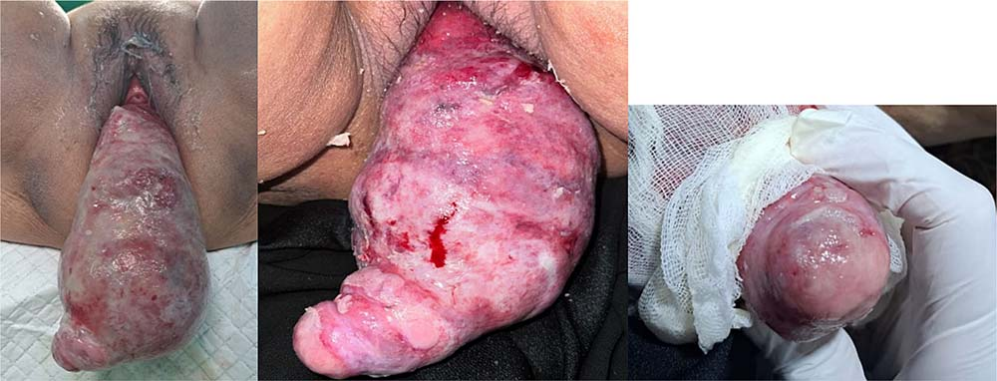
Uterine inversion with submucosal leiomyoma on the corpus. The mass was found to be eroded and covered with slough. A point-like structure was identified at the apex of the mass, suspected of being the tubal ostium; however, the opening of the cervical canal was not identified.

On examination, she was hypertensive and mildly tachycardic. No abdominal mass was identified. Her gynecology evaluation revealed a large pink mass with erosion and white plaque measuring around 30 × 10 × 8 cm (Fig. 1). No cervix-like structure or external uterine ostium was identified, but a point-like structure was identified on the apex, suspected to be the tubal opening. The mass consisted of a cystic and solid part. We suspected that it was a submucosal leiomyoma that had degenerated with cystic parts. Abdominal ultrasound revealed no uterus-like structure and visualized the bladder. The patient was diagnosed with NPUI and submucosal leiomyoma, and a hysterectomy was planned after stabilizing the blood sugar level.

**Figure 2:**
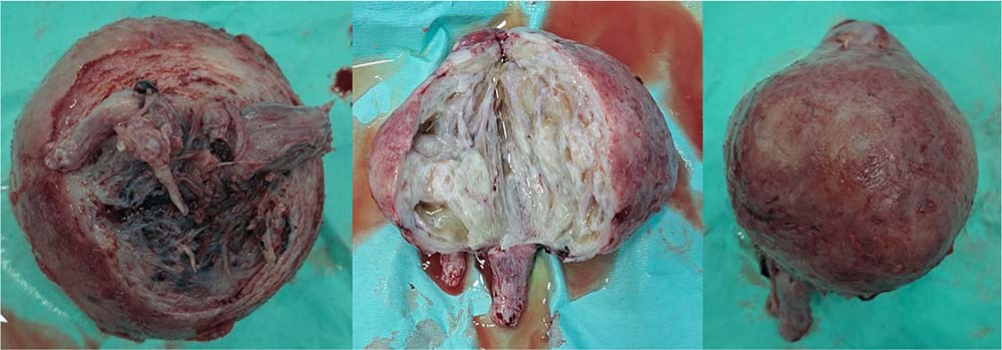
Submucous leiomyoma that was extracted from the uterus. It showed a cystic white degeneration with serous fluid inside.

**Figure 3:**
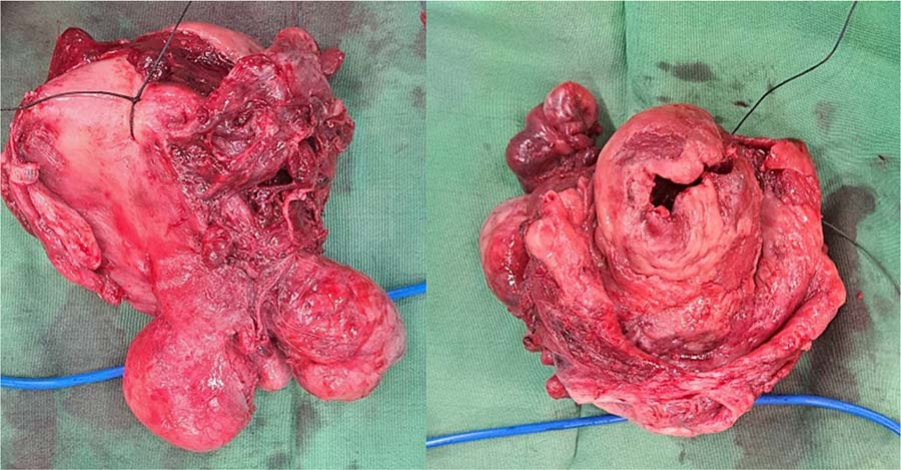
The uterus with the cervix. The corpus was already dissected and excised from the submucous leiomyoma.

Within the operation, a urinary catheter was inserted and the bladder location was identified. We pushed the mass cranially to reposition the uterus. During repositioning, the cyst mass was ruptured and released serous fluid, exposing the uterine cavity. Another team had started to do a laparotomy and performed a median incision. Intraabdominally, we found a vortex-like structure that had sucked the uterus outside, with a twisted round ligament. We decided to incise the cystic mass to decrease the uterus size, and a sondage was then inserted into the cavity to the cervix inside the abdomen. Then, we made a large bundle of gauze in the sondage handle. We decided to do a double approach to reposition the uterus. The sondage was pulled abdominally and pushed vaginally. Soon, the uterus was reversed, and hysterectomy was performed (Figs 2 and 3). The operation was without complication, with 300 cc of bleeding. Postoperatively, the patient had a good recovery. However, a week after discharge, the wound was not healing properly, with some serous fluid coming out of the wound. A superficial surgical site infection was suspected and managed accordingly, with joint care with internal medicine for her diabetes. The histopathological result was angiomatous leiomyoma submucosum, and no further evaluation was needed.

## Discussion

NPUI is an extremely rare case, accounting for 16% of all uterine inversion cases. Common symptoms are abdominal pain, foul vaginal discharge, irregular vaginal bleeding, and sometimes a mass protruding through the vaginal canal. Since the symptoms correlate with the degree of inversion, uterine inversion is classified into four stages (Table 1) [5]. Stages three and four are easier to diagnose since the mass is protruding through the vulva. However, in stages one and two, a high clinical suspicion is needed to diagnose. In this case, the uterine inversion corresponded to stage four uterine inversion, since all of the uterus protruded through the vaginal canal with some part of the vagina everted. A high degree of uterine inversion may lead to some complications, such as urinary retention or even hypovolemic shock due to bleeding from the uterine fibroid [6, 7].

**Table 1:** Uterine inversion classification into four stages, with the uterus protruding through the vulva in stages three and four

Uterine inversion	
Stage 1	Incomplete uterine inversion occurs when the fundus stays inside the uterine cavity.
Stage 2	The uterus fundus was inverted completely through the cervical canal.
Stage 3	Total inversion involves all of the fundus extending outward through the vulva.
Stage 4	The vagina is also everted through the vulva along with an inverted uterus.

Lascarides *et al.* described three clinical signs of uterine inversion: absence of a cervical ring in the proximal part of the mass, no uterine ostium, and rectal examination confirming the absence of the uterus [8]. In acute cases, the tubal ostia may be visible, confirming the diagnosis. Nevertheless, the ostia are usually covered by slough or exudate. Imaging is not essential in diagnosing NPUI, since clinical assessment is usually sufficient. In some cases, ultrasound could help to diagnose potentially malignant cases, and magnetic resonance imaging (MRI) could help to detect metastasis in lymph nodes [9].

The etiology of NPUI was multifactorial, with uterine leiomyoma accounting for 69.5% of the cases, followed by 6.9% carcinoma and 5.6% sarcoma [3]. NPUI can occur when a uterine fibroid grows large enough to distend and irritate the myometrium, provoking contractions and cervical dilation. Thus, expulsing the mass from the uterine cavity pulls the uterine fundus outside. The mechanism can be enhanced by increasing intraabdominal pressure, due to sneezing and coughing, or in this case, straining due to constipation.

Treatment was based on the reproductive desire of the patient, etiology, and the viability of the tissue. In most cases, a total hysterectomy is performed to excise the uterus and the leiomyoma. Repositioning of the uterus could be done vaginally (Spinelli and Kustner techniques) and abdominally (Haultain, Ocejo, Huntington, Tjalma, Sharma). The techniques are described in Table 2 [4, 10, 11]. In our case, both approaches were used. In NPUI, the repositioning is harder than in the puerperial period, since the cervix was constricted and usually accompanied by uterine masses. In this case, we decreased the uterus size by incising the mass, and we modified the sondage handle by making it larger using multiple gauzes rolled to the handle. Pulling the uterine sondage abdominally while pushing it vaginally repositioned the uterus successfully. There is no literature showing pregnancy following successful repositioning in conservative management [4]. There is always a risk of uterine rupture after uterus repositioning in pregnant patients.

**Table 2:** Techniques to reposition uterus vaginally or abdominally

Procedure	
Vaginal repositioning	
Spinelli	Incise the anterior part of the cervix to reposition
Kustner	Incise the posterior part of the uterus and the cervix to reach the cavum douglas vaginally and then reinvert the uterus
Abdominal repositioning	
Haultain	Incision to the posterior surface to release the constriction ring
Ocejo	Incision to the anterior surface to release the constriction ring (be careful of the bladder)
Huntington	Reinvert the uterus by clamping and pulling both round ligaments
Tjalma	Dissect the ureter and the uterine arteries before repositioning and performing the hysterectomy
Sharma	Incise the posterior constriction ring and pull the round ligament; vaginally the fundus was pushed

## Conclusion

NPUI is a rare entity of a disease, commonly associated with submucosal leiomyoma. A high clinical suspicion is necessary to diagnose NPUI since the symptoms are nonspecific. On physical examination, an empty uterus on rectal touche examination, identification of a fallopian tube opening within the vaginal mass, and the absence of a cervical ring confirmed the diagnosis of uterine inversion. Ultrasound could help to diagnose, especially in the early stages of uterine inversion. In NPUI, most management is done with total hysterectomy; however, some cases are conservatively managed, with an unknown prognosis for reproductive function.

## References

[1] Abubeker FA, Misgina M, Ebabu A, et al. Management of nonpuerperal uterine inversion using a combined vaginal and abdominal approach. Case Rep Obstet Gynecol 2020;2020:8827207. 10.1155/2020/8827207 33489392 PMC7794039

[2] Jones HW Jr . Non-puerperal inversion of the uterus. Am J Surg 1951;81:492–5. 10.1016/0002-9610(51)90268-1 14829695

[3] Bruno R, Fernanda D, Maria L. Non-puerperal uterine inversion: a systematic review. Gynecol Obstet Invest 2018;83:428–36.

[4] Shasindran R, Dharshini N, Aruku N, et al. Exploring non-puerperal uterine inversion: a case series. Cureus 2024;16:e53071. 10.7759/cureus.53071 38410337 PMC10896673

[5] Skinner GN, Louden KA. Non-puerperal uterine inversion associated with an atypical leiomyoma. Aust N Z J Obstet Gynaecol 2001;41:100–1. 10.1111/j.1479-828x.2001.tb01304.x 11284635

[6] Perelmuter S, Lin JF, Schiffman M, et al. Rare case of nonpuerperal complete uterine inversion. J Minim Invasive Gynecol 2025;32:406–8. 10.1016/j.jmig.2024.09.023 39326840

[7] Kesrouani A, Cortbaoui E, Khaddage A, et al. Characteristics and outcome in non-puerperal uterine inversion. Cureus 2021;13:e13345. 10.7759/cureus.13345 33754086 PMC7971731

[8] Lascarides E, Cohen M. Surgical management of nonpuerperal inversion of the uterus. Obstet Gynecol 1968;32:376–81. 5754584

[9] Herath RP, Patabendige M, Rashid M, et al. Nonpuerperal uterine inversion: what the gynaecologists need to know? Obstet Gynecol Int 2020;2020:8625186. 10.1155/2020/8625186 32565821 PMC7285247

[10] Sharma NRK, Sharma A, Sharma S. A unique case of recurrent uterine inversion requiring double reposition. J Reprod Contracept Obstet Gynecol 2013;2:427–9. 10.5455/2320-1770.ijrcog20130935

[11] Tjalma WA, Naik R, Monaghan JM, et al. Uterine inversion by a mixed Mullerian tumor of the corpus. Int J Gynecol Cancer 2003;13:894–7. 10.1111/j.1525-1438.2003.13626.x 14675330

